# Metformin Promotes Osteogenic Differentiation by Inducing Autophagy *via* AMPK/mTOR/p70S6K Signaling Pathway in Osteoblasts

**DOI:** 10.2174/0118715303464388260430144310

**Published:** 2026-05-17

**Authors:** Tingting Ye, Liujin Lu, Liuqing Guo, Min Liang

**Affiliations:** 1 Department of Geriatric Endocrinology and Metabolism, The First Affiliated Hospital of Guangxi Medical University, Nanning, Guangxi 530021, China;; 2 Emergency Department, Guangxi Hospital Division of The First Affiliated Hospital, Sun Yat-sen University, Nanning, Guangxi 530022, China

**Keywords:** Metformin, osteoblast, differentiation, autophagy, adenosine 5’-monophosphate-activated protein kinase, 3-methyladenine (3-MA)

## Abstract

**Introduction:**

Metformin has unique application potential in bone metabolism, but the effects of metformin on osteoblasts and its mechanism have not been elucidated.

**Objective:**

The objective of this study was to explore the effects of metformin on osteoblasts and the mechanism underlying these effects.

**Methods:**

Osteoblasts were treated with metformin, and their effects on osteogenic differentiation and autophagy were monitored. 3-methyladenine (3-MA) was used to investigate the relationship between autophagy and metformin-mediated osteogenic differentiation. Additionally, adenosine 5’-monophosphate-activated protein kinase (AMPK) expression was altered to evaluate the involvement of AMPK/ mammalian target of rapamycin (mTOR)/ phosphoprotein 70 ribosomal protein S6 kinase (p70S6K) signaling in the effects of metformin.

**Results:**

Our results revealed that metformin promoted osteogenic differentiation, enhanced autophagy, and activated the AMPK pathway. Treatment with 3-MA inhibited the effects of metformin on osteogenic differentiation, autophagy, and AMPK/mTOR/p70S6K signaling in osteoblasts. The effects of AMPK overexpression were similar to those of metformin on osteoblast autophagy and osteogenic differentiation, whereas AMPK deficiency reversed these effects.

**Discussion:**

Our results reveal the positive effects of metformin on bone metabolism and the underlying mechanisms, which require further validation through *in vivo* experiments and clinical trials.

**Conclusion:**

Our findings revealed that AMPK/mTOR/p70S6K signaling is involved in metformin-regulated osteogenic differentiation through the induction of autophagy.

## 1 INTRODUCTION

Osteoporosis is a metabolic skeletal disorder characterized by the loss of bone mass and deterioration of bone microarchitecture, leading to increased fracture risk [1]. First-line therapies such as bisphosphonates primarily inhibit bone resorption and prevent further bone loss but do not improve bone formation and may even induce osteoblast apoptosis [2]. Additionally, the use of bisphosphonates can cause side effects such as upper gastrointestinal symptoms, an acute phase response, osteonecrosis of the jaw, renal injury, and atypical femur fractures [3]. Thus, safer and more potent osteogenic medications are needed for osteoporosis treatment.

Metformin, which is traditionally used to treat type 2 diabetes, has few adverse effects and has been reported to be advantageous for bone metabolism [4-6]. However, some studies have shown that metformin has no effect on bone metabolism or is even harmful to this process [7, 8]. A complete understanding of the effect of metformin on bone and its underlying mechanism remains elusive.

Autophagy is a core molecular pathway that maintains cellular and organismal homeostasis [9]. The upregulation of autophagy in osteoblasts enhances differentiation and mineralization and increases the expression of key transcription factors involved in osteoblast function, thus ameliorating osteoporosis [10]. Accumulating evidence has indicated that autophagy is a key modulator of the pharmacological activity of metformin.

The effects of metformin on autophagy are mediated by multiple signaling pathways, including AMPK-related signaling pathways [11]. AMPK serves as a crucial sensor of cellular energy to modulate energy equilibrium and metabolism [12]. The activation of AMPK increases catabolism and decreases anabolism in the cells through the phosphorylation of the essential protein mTOR [13]. mTOR, which senses nutrients in contrast to AMPK, promotes anabolism and increases cell growth through the phosphorylation of substrates such as p70S6K. AMPK is activated in response to energy or nutrient shortage, which inactivates mTOR and p70S6K, thus inducing autophagy in cells [14]. Numerous studies have revealed the effects of metformin on AMPK activity [15-18] and the osteogenic effects of AMPK activation [19-23]. In addition, the mTOR/p70S6K signaling pathway is involved in cellular proliferation, differentiation, and autophagy [24]. In a previous study [25], we reported that metformin protected against dexamethasone-induced apoptosis in osteoblasts through AMPK/mTOR/p70S6K signaling. However, under physiological conditions, the involvement of the AMPK pathway in metformin-induced osteogenesis has not been fully elucidated. In prior work, we focused on osteoblast apoptosis, but osteogenic differentiation was not examined. To our knowledge, investigations into the involvement of AMPK/mTOR/p70S6K signaling in the effects of metformin on osteogenic differentiation and autophagy in osteoblasts are limited.

Therefore, this study was designed to investigate the effects of metformin on osteogenic differentiation and autophagy in osteoblasts and to explore whether AMPK/mTOR/p70S6K signaling is involved in these effects.

## 2 MATERIALS AND METHODS

### 2.1 Animals and Ethics Statement

This study was an *in vitro* study. Ten male and ten female Sprague–Dawley (SD) rats (weight 7.6± 1.3 g) born within 24 hours(h) that were housed in a specific pathogen-free laboratory at 20–26 °C with 40–70% humidity and a 12 h light/dark cycle were purchased from the Laboratory Animal Center of Guangxi Medical University. The animal treatment protocols were thoroughly reviewed and approved by the Ethical Review Committee for the First Affiliated Hospital of Guangxi Medical University (Approval ID:2022-E472-01).

### 2.2 Reagents and Antibodies

Metformin (HY-17471A) and 3-MA (HY-19312) were obtained from Med Chem Express. Primary antibodies against LC3 (ab48394), Beclin1 (ab207613), p62 (ab109012), and alkaline phosphatase (ALP) (ab65834) were obtained from Abcam. Antibodies specific to collagen I (COL I) (91144), AMPK (2532), p-AMPK (2531), mTOR (2972), p-mTOR (5536), p70S6K (9202), and p-p70S6K (9205) were procured from Cell Signaling Technology. GAPDH (D110016) was sourced from Sangon Biotech in China. Goat anti-rabbit IgG(H+L) secondary antibody (SA5-35571) was obtained from Invitrogen. Coralite594-conjugated goat anti-rabbit IgG(H+L) (SA00013-4) was acquired from Proteintech (China).

### 2.3 Isolation, Culture, and Identification of Primary Osteoblasts

As previously described [26], primary osteoblasts were isolated from the calvaria of neonatal SD rats. Primary osteoblasts were cultured in Dulbecco’s modified Eagle’s medium (Gibco) supplemented with 10% fetal bovine serum (Gibco) and 1% penicillin/streptomycin/amphotericin B (Solarbio, China) and kept in a humidified incubator (Thermo, USA) at 37 °C in 5% CO_2_. To identify osteoblasts, the cells were washed three times with PBS and fixed in 4% paraformaldehyde for 15 minutes(min) at room temperature. ALP staining was subsequently performed using a 5-bromo-4-chloro-3-indolylphosphate / nitro blue tetrazolium ALP color development kit (Beyotime, China), and alizarin red staining was carried out using 0.2% alizarin red S solution (Solarbio, China). All cell experiments were repeated three times independently using osteoblasts from different passages.

### 2.4 Cell Viability Assay

Osteoblasts were cultured in 96-well plates supplemented with varying concentrations of metformin (50, 100, 200, 400, 800,1600, and 3200 μM) at a density of 3×104 cells/well for 24, 48, 72, and 96 h. Cell proliferation was assessed using the Cell Counting Kit-8 (CCK8) (Melunbio, China), as suggested by the manufacturer’s protocols.

### 2.5 ALP Activity Assay

After being washed thrice with PBS, the cells were lysed in RIPA lysis buffer without inhibitors (Beyotime, China) and then centrifuged to obtain the supernatant. ALP activity was evaluated using an ALP Assay Kit (Beyotime, China) according to the manufacturer’s instructions.

### 2.6 Transmission Electron Microscopy (TEM)

Following treatment with metformin, osteoblasts were fixed in 2.5% glutaraldehyde at 4°C. The samples were then pre-embedded in agarose, post-fixed, infiltrated with resin, and polymerized. Ultrathin sections (60–80nm) were prepared using an ultramicrotome, stained with 2% uranyl acetate in ethanol (protected from light) for 8min, and then with 2.6% lead citrate (in a CO_2_-free environment) for 8min. Autophagosomes were observed using a TEM (Hitachi, Japan).

### 2.7 Lentivirus Transfection

Lentivirus-AMPK overexpressing AMPK, short hairpin RNA (shRNA)-AMPK suppressing AMPK expression, and corresponding negative controls (NC) were designed and produced by Genechem (China). Cells were transfected with different lentiviral vectors following the manufacturer’s instructions. After 72 h of transfection, the culture medium was replaced with fresh medium containing 4μg/ml puromycin to establish stably transfected cell lines.

### 2.8 Western Blotting (WB)

We lysed cells using RIPA lysis buffer (Beyotime, China) supplemented with 1% phenylmethanesulfonyl fluoride, and measured the protein concentration with a BCA protein assay kit (Beyotime, China). Equal amounts of protein were resolved by 8%, 10% or 12% SDS–PAGE. The samples were transferred to a polyvinylidene fluoride (PVDF) membrane. The membranes were cut horizontally. Appropriate primary antibodies were used to detect individual proteins after blocking with 5% skim milk. The proteins were incubated with a goat anti-rabbit IgG (H+L) secondary antibody for 1 h at room temperature. An Odyssey system (LICOR Odyssey, USA) was used to visualize the proteins on the PVDF membranes.

### 2.9 Immunofluorescence Staining

Cells were cultured with different treatments, fixed with 4% paraformaldehyde for 20 min, and then permeabilized with 0.2% Triton X-100 for 10 min. We blocked the cells with 1% bovine serum albumin (BSA) for one hour, replaced the BSA solution with primary antibodies, and incubated them overnight at 4 °C. After washing with PBS, the cells were incubated with Coralite594-conjugated goat anti-rabbit IgG (H+L) secondary antibody. Following DAPI (Solabrio, China) staining, images were acquired using a fluorescence microscope (Olympus, Japan).

### 2.10 Statistical Analysis

The experimental data are presented as the means ± standard deviations (SDs). Data analysis was performed using one-way ANOVA, followed by Tukey’s post hoc test in SPSS 26.0 software, with *P*<0.05 considered significant. All the experiments were repeated at least three times.

## 3 RESULTS

### 3.1 Metformin Regulates Osteoblast Viability and Osteogenic Differentiation

The effect of metformin on cell viability was evaluated *via* a CCK8 assay. An initial screen revealed 50–3200 μM and 24–96 h to be the optimal concentration and exposure time, respectively, for the treatment of osteoblasts with metformin *in vitro*. The optimal treatment conditions included 800 μM metformin for 72 h (Fig. **1A**).

COL I, a specific osteogenic marker constituting more than 90% of the organic bone extracellular matrix [27], and ALP, an early differentiation marker that facilitates bone mineralization [28], were analyzed by ALP activity, WB, and immunocytochemistry. The osteoblasts were treated with metformin (50, 200, 800, 1600, or 3200 μM) for 72 h. ALP activity in the osteoblasts significantly increased in a dose-dependent manner upon treatment with 50, 200, and 800 μM metformin but was suppressed by treatment with 3200 μM metformin (Fig. **1B**). Compared with the control group, treatment with metformin at concentrations of 50, 200, 800, and 1600 μM increased ALP protein expression, and ALP protein expression was greatest in the group treated with 800 μM metformin (Fig. **1C-F**). A significant increase in COL I protein expression was noted in the groups treated with 200, 800, and 1600 μM metformin, whereas treatment with 3200 μM metformin decreased COLI protein expression (Fig. **1C** and **D**). Afterward, the cells were treated with 800 μM metformin for 48, 72, or 96 h. Compared with control cells, metformin-treated cells displayed substantially greater ALP activity (Fig. **1G**) and ALP protein expression, with maximum values observed at 72 h (Fig. **1H-K**). Compared with that in the control group, COL I protein expression clearly increased in the groups treated with metformin for 48 or 72 h (Fig. **1H** and **I**). These data suggest that treatment with metformin at a specific concentration for a specific duration promotes osteogenic differentiation.

### 3.2 Metformin Induces Autophagy in Osteoblasts

To explore the mechanism by which metformin induces osteogenic differentiation, we used TEM, WB, and immunofluorescence staining to monitor changes in autophagy.

A significant increase in the number of autophagosome was observed in the groups treated with 200 and 800 μM metformin (Fig. **2A**). Additionally, treatment with metformin at these concentrations resulted in significant increases in the LC3 II/I ratio (Fig. **2B**-**E**). Increased Beclin1 levels and decreased p62 levels were detected in the groups treated with 50, 200, and 800 μM metformin, whereas the opposite effects were detected in the group treated with 3200 μM metformin (Fig. **2B** and **C**)

A time course experiment revealed that metformin significantly increased the number of autophagosome and the LC3 II/I ratio at only 72 h (Fig. **2F**-**J**). Notably, Beclin1 levels increased and p62 levels decreased in the groups treated for 48 and 72 h compared with those in the control group (Fig. **2G** and **H**). These findings suggest that treatment with a specific concentration of metformin for a specific duration increases autophagy in osteoblasts.

### 3.3 3-MA Inhibits Autophagy, Osteogenic Differentiation, and Ampk Signaling in Osteoblasts

The autophagy inhibitor 3-MA is a potent PtdIns3K inhibitor that effectively blocks early-stage autophagy [29]. 3- MA was used to clarify whether metformin stimulates osteogenic differentiation by inducing autophagy in osteoblasts.

Compared with the control treatment, the 3-MA treatment suppressed autophagy and decreased the expression of osteogenic differentiation-related proteins (ALP and COL I), but these parameters normalized with additional metformin treatment (Fig. **3A**-**C**). These results were further substantiated by immunofluorescence staining (Fig. **3D**-**F**). WB also revealed that 3-MA inhibited AMPK/mTOR/p70S6K signaling (Fig. **3G** and **H**). These data suggested that the stimulation of osteogenic differentiation and the activation of AMPK signaling in osteoblasts induced by metformin were largely reversed by the suppression of autophagy induced by 3-MA treatment.

### 3.4 Role of AMPK Signaling in Metformin-stimulated Osteogenic Differentiation Through the Induction of Autophagy in Osteoblasts

We hypothesize that metformin enhances osteogenic differentiation through the induction of autophagy, possibly *via* AMPK/mTOR/p70S6K signaling. To further support our hypothesis, we upregulated and downregulated AMPK expression *via* lentivirus transduction.

WB (Fig. **4A**-**C**) and immunofluorescence staining (Fig. **4D**-**F**) revealed that both autophagy (as shown by increases in the LC3 II/I ratio and Beclin1 level and a decrease in the p62 level) and osteogenic differentiation (as shown by increases in ALP and COLI levels) were enhanced when AMPK expression was upregulated (Fig. **4G** and **H**) compared with those in the control group, which is in line with the effects of metformin. When cells were both transduced with AMPK-expressing lentivirus and treated with metformin, autophagy and osteogenic differentiation increased to a greater extent than they did when cells were treated with metformin alone. In summary, the ability of AMPK upregulation to induce autophagy and osteogenic differentiation in osteoblasts was consistent with the effects of metformin, and AMPK upregulation and metformin treatment had synergistic effects.

Furthermore, we downregulated AMPK expression *via* shRNA transfection. Compared with the control treatment, AMPK-specific knockdown resulted in decreases in the LC3 II/I ratio and Beclin1, ALP, and COL I levels, and an increase in the p62 level, and these changes were largely reversed by treatment with additional metformin (Fig. **5A**-**C**). Immunostaining (Fig. **5D**-**F**) for LC3 and ALP confirmed robust decreases in LC3 and ALP staining intensity after AMPK was knocked down by shRNA-AMPK transfection, but no difference in staining intensity was observed when shRNA-AMPK and metformin were applied together. AMPK knockdown subsequently increased the p-mTOR/mTOR and p-p70S6K/p70S6K protein ratios (Fig. **5G** and **H**). These data showed that the downregulation of AMPK expression significantly inhibited osteogenic differentiation and autophagy in osteoblasts and reversed metformin-induced osteogenic differentiation and autophagy.

## 4 DISCUSSION

With the aging of the global population, osteoporosis has emerged as a critical public health issue. Osteoporosis is characterized by a bone remodeling imbalance that results from either increased bone resorption mediated by osteoclasts or decreased bone formation mediated by osteoblasts. The primary functional cells involved in bone formation, osteoblasts, are responsible for producing, secreting, and mineralizing the bone matrix and can transform or differentiate to form new bone [30].

Metformin remains a first-line antidiabetic drug but has shown new potential in other areas, such as cancer, aging, and bone disorders [31-34]. The osteogenic effects of metformin have mostly been validated, but the results have been inconsistent. A double-blind, randomized, placebo-controlled trial revealed that overweight diabetic patients treated with metformin experienced an approximately 0.7% increase in appendicular bone mineral content and a 0.5% increase in bone mineral density after 18 months of treatment, whereas those patients who received a placebo showed decreases in these parameters [35]. An animal study further revealed that, compared with a control treatment, metformin treatment led to substantial increases in ALP, osteocalcin, and calcium levels in diabetic rats [36]. In the hyperglycemic osteoblastic cell line MC3T3-E1, metformin significantly increased ALP and osteocalcin secretion and decreased apoptosis [37]. Nevertheless, according to a two-year clinical trial, patients who received metformin alone and those who received metformin and rosiglitazone showed 13 and 21% reductions in the procollagen I N-terminal propeptide (PINP) concentration, respectively [38]. A study by Jeyabalan *et al.* also revealed that metformin had no effect on bone resorption but did reduce the rate of bone formation in the trabecular bone of female mice following ovariectomy and had no effect on fracture healing in female Wistar rats [39]. The inconsistency of these results might be due to differences in the inclusion and exclusion criteria applied to the subjects, the experimental models used, and the metformin concentrations applied. Our study revealed that metformin promotes osteogenic differentiation, which is consistent with the results of most related studies.

In mouse calvarial osteoblasts and MC3T3-E1 cells, treatment with 800 μM metformin resulted in the activation of AMPK phosphorylation and upregulation of osteoprotegerin (OPG) expression [40]. However, in another study, 2 mM metformin treatment caused sustained AMPK phosphorylation and reduced the mRNA expression of osteogenic differentiation markers, ALP activity, and mineralized nodules, leading to inhibited osteogenic differentiation [41]. These results led us to conclude that metformin has different effects on osteoblasts depending on its concentration. Thus, in this study, osteoblasts were incubated with 50–3200 μM metformin, and changes in proliferation and osteogenic differentiation were investigated. At lower doses (50, 200, and 800 μM), metformin promoted proliferation and osteogenic differentiation, whereas at a higher dose (3200 μM), metformin inhibited these processes; this difference in the effects of metformin may have occurred because of toxicity [42]. For the optimal treatment of osteoblasts, 800 μM metformin was applied for 72 h. These findings are consistent with the findings of clinical practice that metformin has an appropriate dose range beyond which its efficacy does not increase or can even decrease [43]. Accurate optimization of metformin dose and treatment duration is crucial for achieving the maximum effect of metformin on bone health. These findings will facilitate the therapeutic application of metformin for osteoporosis.

Metformin can induce autophagy. Enhanced mitophagy in individuals with type 2 diabetes who received metformin resulted in improved mitochondrial morphology and function in peripheral blood mononuclear cells, which may have mitigated diabetes-related complications [44]. Moreover, metformin significantly increased autophagy and mitochondrial bioenergetics concomitant with the attenuation of Th17 inflammation, thus mitigating aging-related inflammation [45]. Autophagy, a major catabolic process in eukaryotic cells that is responsible for the degradation and recycling of damaged organelles and macromolecules, is upregulated during osteogenic differentiation and mineralization and plays a crucial role in maintaining bone homeostasis [46]. Increased autophagy during osteoblastic osteogenic differentiation stimulated by metformin was also observed in this study. Metformin may promote osteogenic differentiation by inducing autophagy. These findings were confirmed by our data, which revealed that 3-MA attenuated metformin-mediated autophagy and blocked the ability of metformin to promote osteogenic differentiation of osteoblasts. These results are in agreement with those of most related studies [47-49].

We further investigated the pathway through which metformin induces autophagy to promote osteoblast osteogenic differentiation. Most available data indicate that metformin directly affects osteoblastic proliferation and differentiation through AMPK activation [50]. The ability of metformin to affect the expression of key osteogenic genes, including ALP, osteocalcin, bone sialoprotein, and small heterodimeric partner proteins in MC3T3E1 cells was attenuated by compound C (an inhibitor of AMPK) [51]. AMPK, which is pivotal to the mechanism of metformin activity, also affects autophagy. Metformin simultaneously activated AMPK and inhibited mTOR, leading to the inhibition of p70S6K activity and the induction of autophagy [52]. Our results also revealed that metformin activated AMPK/mTOR/p70S6K signaling to promote osteogenic differentiation and autophagy in osteoblasts. In addition, 3-MA inhibited the expression of AMPK signaling proteins in our study. This effect could be attributed to increased oxidative stress caused by nuclear factor erythroid 2-related factor 2 (Nrf2) activation induced by the inhibition of autophagy, which in turn suppressed AMPK phosphorylation [53-56]. These findings suggest that metformin may stimulate the differentiation of osteoblasts through the induction of autophagy *via* the AMPK signaling pathway. To confirm this, we used lentiviruses to upregulate or downregulate AMPK expression. In our study, the overexpression of AMPK facilitated osteoblastic autophagy and osteogenic differentiation and enhanced the effects of metformin on osteoblasts. Conversely, AMPK deficiency had an inhibitory effect. These findings suggest that AMPK/mTOR/p70S6K signaling is involved in metformin-mediated regulation of osteoblast osteogenic differentiation through the induction of autophagy (Fig. **6**). Notably, metformin is known for its pleiotropic properties. In addition to affecting bone metabolism, it also modulates immune-inflammatory pathways and macrophage function through AMPK [57-59]. We hypothesize that in addition to its direct action on osteoblasts, metformin indirectly contributes to bone protection by fostering a favorable osteoimmune microenvironment, thus achieving dual regulatory effects. This represents a promising direction for our future research.

## CONCLUSION

The results of this study demonstrate that metformin promotes the osteogenic differentiation of osteoblasts through AMPK/mTOR/p70S6K signaling -induced autophagy and that the concentration of metformin and duration of metformin treatment are relevant to these effects. Our findings provide a theoretical foundation for studies on bone metabolism and the future clinical application of metformin.

## LIMITATIONS

This study has several limitations. First, 800 µM metformin exceeds clinical plasma levels and may elicit nonspecific stress responses, necessitating further validation in animal models and clinical trials. Autophagy was assessed mainly *via* static markers (LC3-II conversion and p62 degradation) without monitoring autophagy flux assays or downstream effectors, highlighting the necessity for more detailed mechanistic exploration. Additionally, while the link between autophagy and osteogenic differentiation was demonstrated using 3-MA, definitive causal evidence would necessitate combining pharmacological effects with genetic modulation of key autophagy genes.

## AUTHORS’ CONTRIBUTIONS

The authors confirm their contributions to the paper as follows: ML contributed to project administration, funding acquisition, supervision, conceptualization, writing, review & editing. TY contributed to methodology, data curation, formal analysis, visualization, investigation, validation, and writing – original draft. LL was involved in methodology, data curation, formal analysis, visualization, investigation, and validation. LG contributed to data curation and formal analysis.

## Figures and Tables

**Fig. (1):**
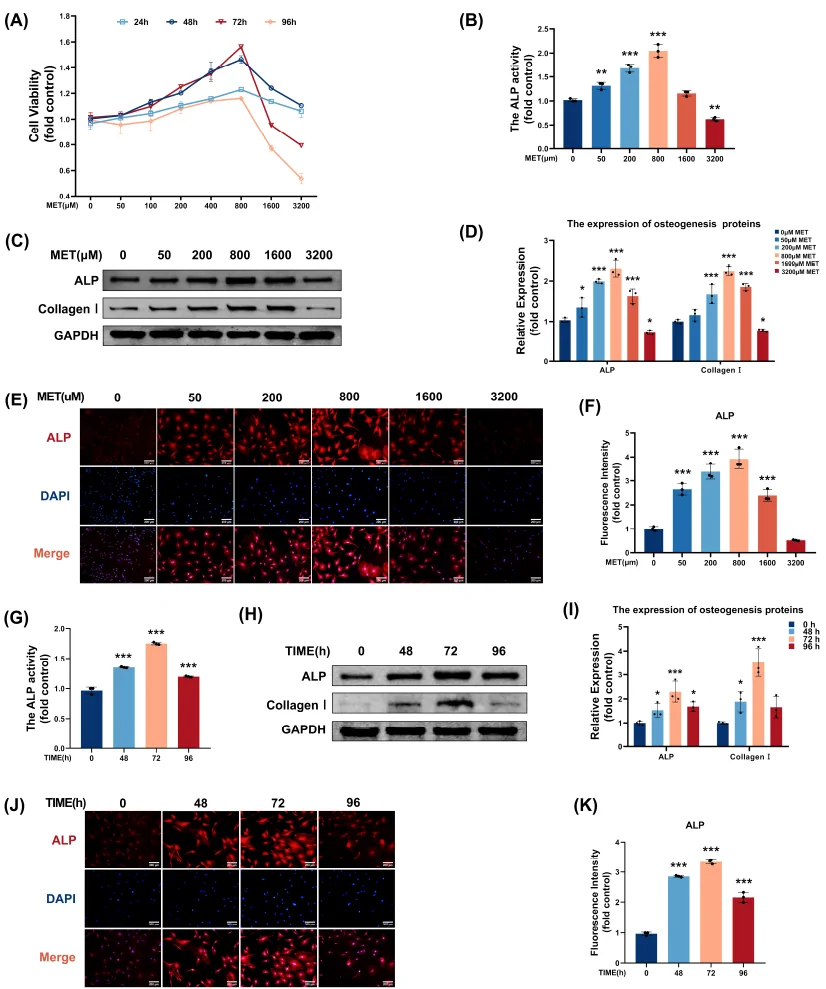
Metformin enhances proliferation and osteogenic differentiation in osteoblasts.**(A)** Cell viability assessed by CCK-8 assay; **(B)** ALP activity after 72 h of treatment with metformin (50–3200 μM); **(C)** WB results and ** (D)** quantitative analysis of osteogenic differentiation-related proteins following metformin treatment (50–3200μM) for 72 h;** (E)** Immunofluorescence staining and** (F)** quantitative analysis of ALP protein expression following metformin treatment (50–3200μM) for 72 h;** (G) **ALP activity after treatment with 800μM metformin for 48–96h; **(H)** WB results and** (I)** quantitative analysis of osteogenic differentiation-related proteins after treatment with 800μM metformin for 48–96h;** (J)** Immunofluorescence staining and** (K)** quantitative analysis of ALP protein expression after treatment with 800μM metformin for 48–96h; * *P*<0.05, ** *P*<0.01, *** *P*<0.001 *versus* the control group.

**Fig. (2):**
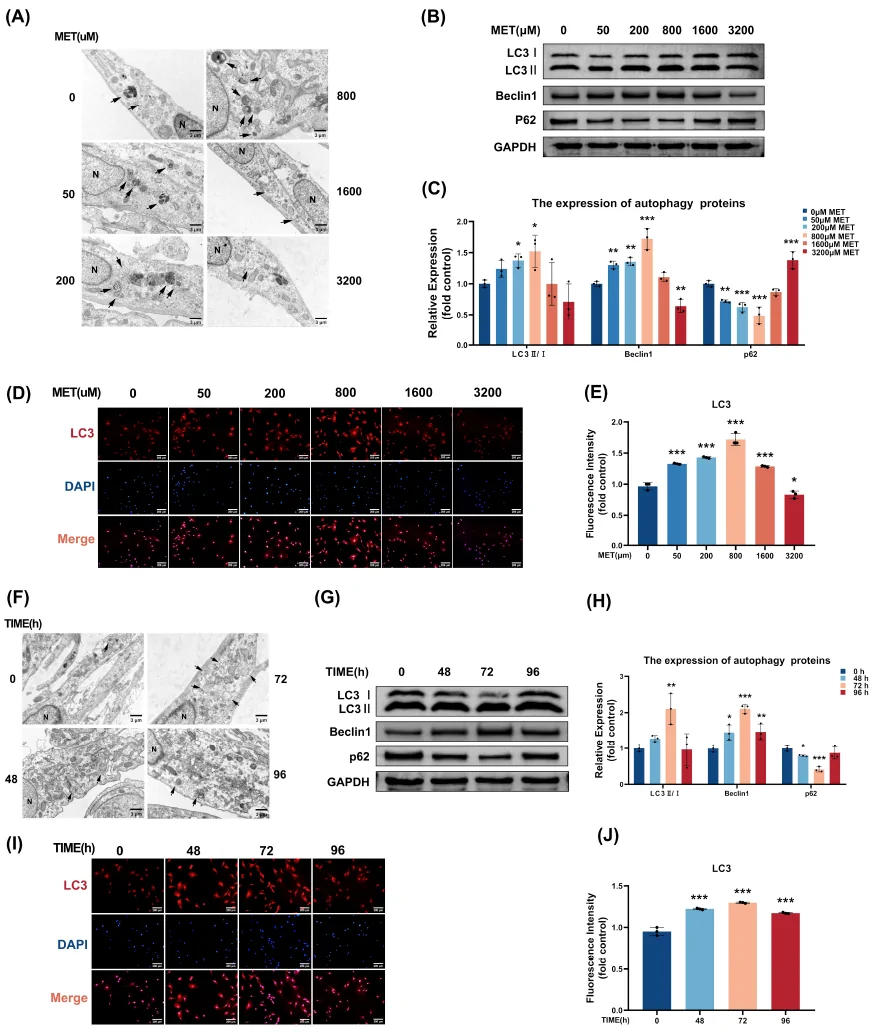
Metformin induces autophagy in osteoblasts.** (A) **Autophagosomes were observed by TEM following treatment with metformin (50–3200μM) for 72 h; N: nucleus; arrows: autophagosomes; **(B)** WB results and** (C)** quantitative analysis of autophagy-related proteins following metformin treatment (50–3200μM) for 72 h; **(D)** Immunofluorescence staining and** (E)** quantitative analysis of LC3 protein following metformin treatment (50–3200μM) for 72 h;** (F)** Autophagosomes were observed by TEM following treatment with 800μM metformin for 48–96h; N: nucleus; arrows: autophagosomes;** (G) ** WB results and** (H)** quantitative analysis of autophagy-related proteins after treatment with 800μM metformin for 48–96h;** (I)** Immunofluorescence staining and** (J) **quantitative analysis of LC3 protein after treatment with 800μM metformin for 48–96h; * *P*<0.05, ** *P*<0.01, *** *P*<0.001 *versus* the control group.

**Fig. (3):**
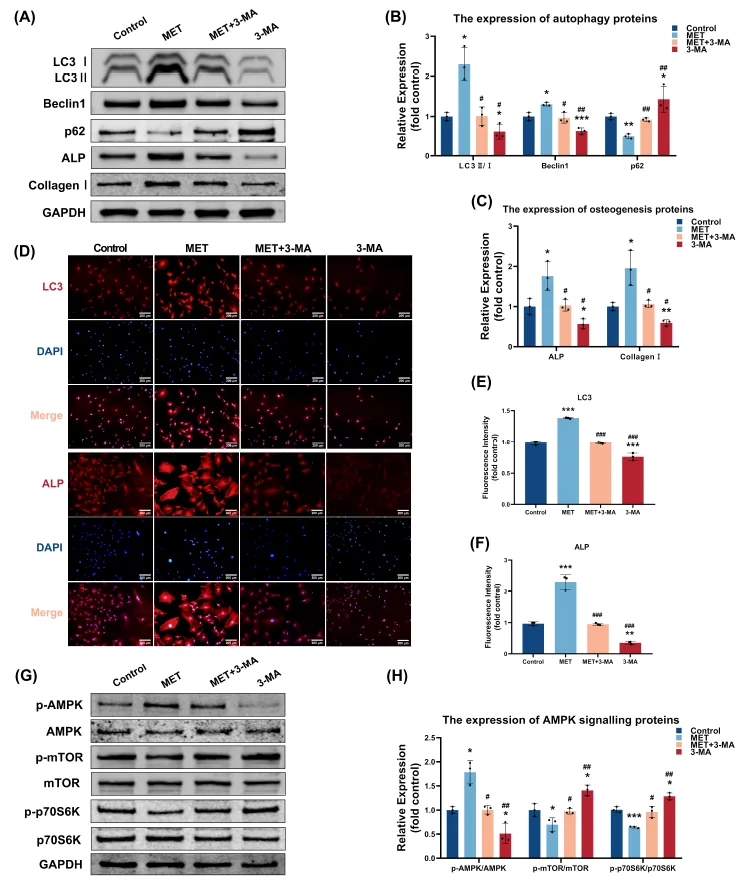
3-MA inhibits autophagy, osteogenic differentiation, and AMPK signaling in osteoblasts. Cells were cultured in 800uM metformin with or without 3-MA for 72 h; **(A)** The expression of autophagy- and osteogenic differentiation-related proteins were assessed by WB; **(B)** Quantitative analysis of autophagy-related protein levels; **(C)** Quantitative analysis of osteogenic differentiation-related protein levels; **(D)** Immunofluorescence staining of LC3 and ALP protein;** (E)** Quantitative analysis of immunofluorescence staining for LC3 protein; **(F)** Quantitative analysis of immunofluorescence staining for ALP protein; **(G)** WB and **(H)** quantitative analysis of AMPK pathway-related proteins; * *P*<0.05, ** *P*<0.01, *** *P*<0.001 *versus* the control group; # *P*<0.05, ## *P*<0.01, ### *P*<0.001 *versus* the metformin group.

**Fig. (4):**
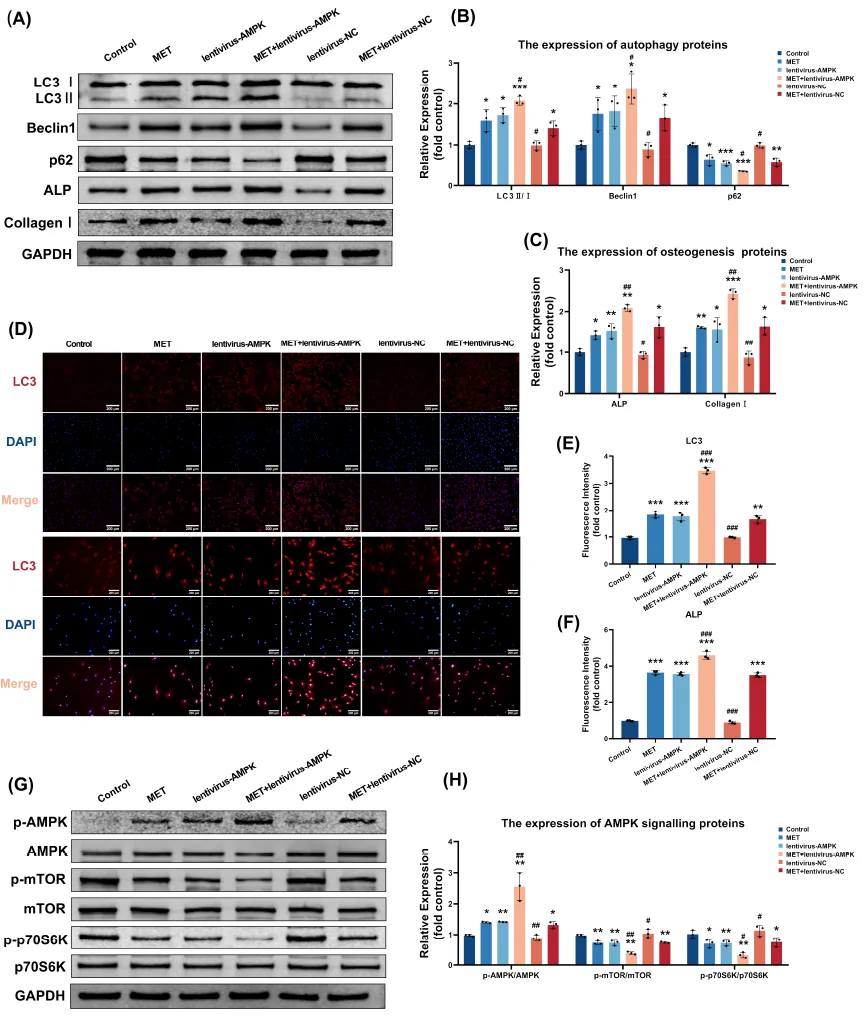
The effects of AMPK upregulation on osteogenic differentiation, autophagy, and AMPK signaling in osteoblasts. Cells were cultured in 800 μM metformin with or without lentivirus-AMPK for 72 h. **(A)** The expression of autophagy- and osteogenic differentiation-related proteins were assessed by WB; **(B)** Quantitative analysis of autophagy-related protein levels; **(C)** Quantitative analysis of osteogenic differentiation-related protein levels; **(D)** Immunofluorescence staining of LC3 and ALP protein;** (E)** Quantitative analysis of immunofluorescence staining for LC3 protein; **(F)** Quantitative analysis of immunofluorescence staining for ALP protein;** (G)** WB and** (H) ** quantitative analysis of AMPK pathway-related proteins; * *P*<0.05, ** *P*<0.01, *** *P*<0.001 *versus* the control group; # *P*<0.05, ## *P*<0.01, ### *P*<0.001 *versus* the metformin group.

**Fig. (5):**
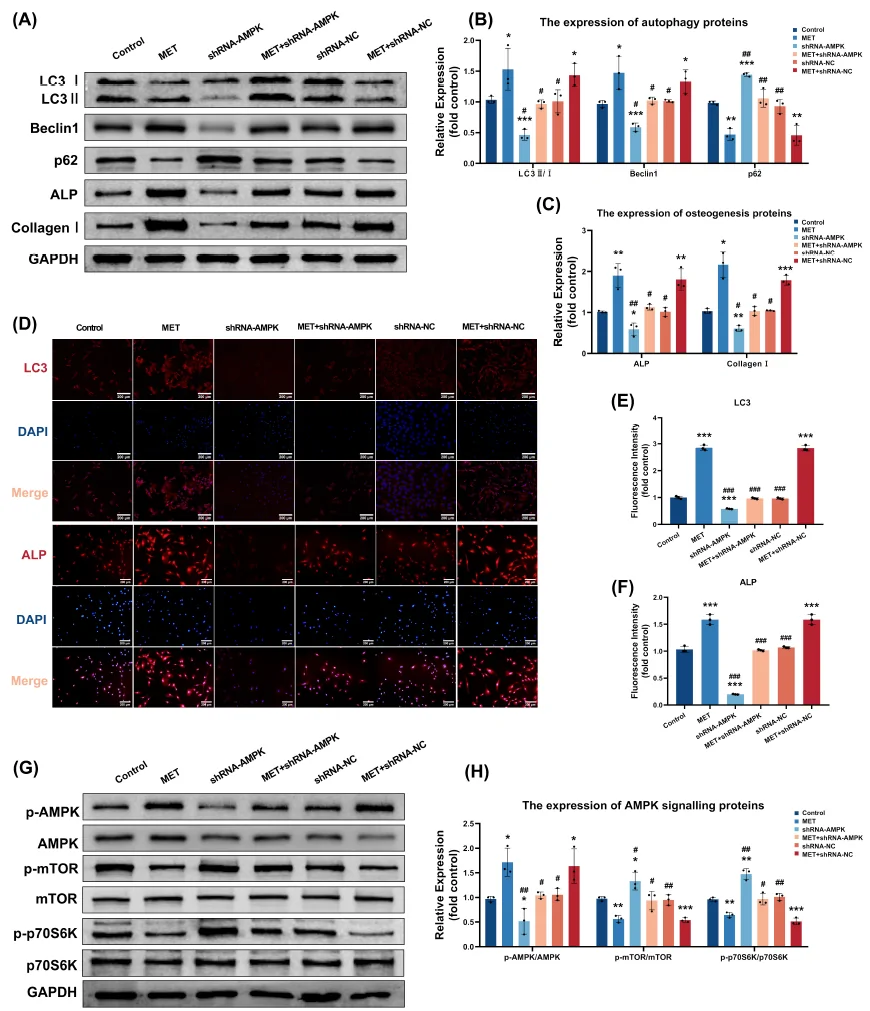
Downregulation of AMPK attenuates the promoted effects of metformin on osteogenic differentiation, autophagy, and AMPK signaling in osteoblasts. Cells were cultured in 800 μM metformin with or without shRNA-AMPK for 72 h. **(A)** The expression of autophagy- and osteogenic differentiation-related proteins were assessed by WB; **(B)** Quantitative analysis of autophagy-related protein levels; **(C)** Quantitative analysis of osteogenic differentiation-related protein levels; **(D)** Immunofluorescence staining of LC3 and ALP protein; **(E)** Quantitative analysis of immunofluorescence staining for LC3 protein;** (F)** Quantitative analysis of immunofluorescence staining for ALP protein; **(G) ** WB and **(H)** quantitative analysis of AMPK pathway-related proteins**;** * *P*<0.05, ** *P*<0.01, *** *P*<0.001 *versus* the control group; # *P*<0.05, ## *P*<0.01, ### *P*<0.001 *versus* the metformin group.

**Fig. (6):**
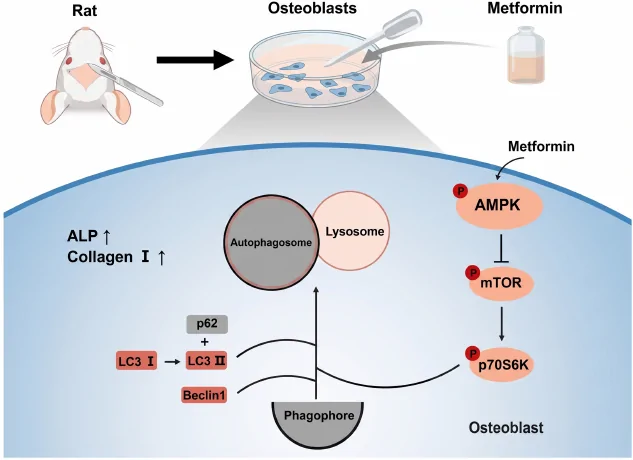
Schematic representation depicting the mechanisms underlying metformin-induced osteogenic differentiation by induction of AMPK signaling- mediated autophagy. The diagram illustrates that metformin activates AMPK in osteoblasts, which subsequently inhibits the phosphorylation of mTOR and p70S6K, thus inducing autophagy (characterized by increased LC3-II conversion and Beclin1 expression, and p62 degradation). Enhanced autophagy promotes osteogenic differentiation, as demonstrated by elevated expression of osteogenic markers such as ALP and collagenI.

## Data Availability

The data generated in the present study may be requested from the corresponding author.

## LIST OF ABBREVIATIONS

AMPK: Adenosine 5’-Monophosphate-Activated Protein Kinase
3-MA: 3-Methyladenine
mTOR: Mammalian Target Of Rapamycin
p70S6K: Phosphoprotein 70 Ribosomal Protein S6 Kinase
ALP: Alkaline Phosphatase
COL I: Collagen I
